# Myocyte enhancer factor 2A promotes proliferation and its inhibition attenuates myogenic differentiation via myozenin 2 in bovine skeletal muscle myoblast

**DOI:** 10.1371/journal.pone.0196255

**Published:** 2018-04-26

**Authors:** Ya-Ning Wang, Wu-Cai Yang, Pei-Wei Li, Hong-Bao Wang, Ying-Ying Zhang, Lin-Sen Zan

**Affiliations:** 1 College of Animal Science and Technology, Northwest A&F University, Yangling, Shaanxi, China; 2 National Beef Cattle Improvement Center in China, Yangling, Shaanxi, P. R. China; 3 Animal Husbandry and Veterinary Research Institute, Shanghai Academy of Agricultural Sciences, Shanghai, P. R. China; 4 National and Provincial Joint Engineering Research Center of Modern Cattle Biotechnology and Applications, Yangling, Shaanxi, P. R. China; Wageningen UR Livestock Research, NETHERLANDS

## Abstract

Myocyte enhancer factor 2A (MEF2A) is widely distributed in various tissues or organs and plays crucial roles in multiple biological processes. To examine the potential effects of MEF2A on skeletal muscle myoblast, the functional role of MFE2A in myoblast proliferation and differentiation was investigated. In this study, we found that the mRNA expression level of *Mef2a* was dramatically increased during the myogenesis of bovine skeletal muscle primary myoblast. Overexpression of MEF2A significantly promoted myoblast proliferation, while knockdown of MEF2A inhibited the proliferation and differentiation of myoblast. RT-PCR and western blot analysis revealed that this positive effect of MEF2A on the proliferation of myoblast was carried out by triggering cell cycle progression by activating CDK2 protein expression. Besides, MEF2A was found to be an important transcription factor that bound to the myozenin 2 (*MyoZ2*) proximal promoter and performed upstream of *MyoZ2* during myoblast differentiation. This study provides the first experimental evidence that MEF2A is a positive regulator in skeletal muscle myoblast proliferation and suggests that MEF2A regulates myoblast differentiation via regulating *MyoZ2*.

## Introduction

Skeletal muscle is an important constituent in indicating muscle growth and livestock muscle quality [1]. Previous studies have demonstrated that the development of skeletal muscle is a complex process that determines the number of muscle fibers, mass, and fiber type [2–5]. At the genetic level, the maintenance of skeletal muscle function mainly depends on myocytes, the primary cellular component of skeletal muscle. Therefore, it is important to understand the molecular mechanisms that regulates skeletal muscle myogenesis.

MEF2A, an evolutionarily conserved transcription factor, is widely distributed in various tissues or organs and play crucial roles in multiple biological processes including cell fate determination, migration and shape [6–9]. Previous studies showed that MEF2A is a key regulator in myogenesis. However, the underlying mechanisms of regulation within the various stages of myogenesis, such as proliferation and differentiation of progenitor cells, has not been fully elucidated [10]. A limited number of studies has suggested that MEF2A may regulate cell proliferation. In mice, global deletion of MEF2A impaired regenerative myogenesis, and knockdown of MEF2A in C2C12 cells severely impaired myotube formation [11–13]. Only one study investigated the function of MEF2A in myoblast proliferation [14]. However, more robust evidence is needed to determine whether and how MEF2A regulates myoblast proliferation and differentiation.

MEF2A has a functional role in the differentiation of muscle cells [9, 13, 14]. Most of the present studies of MEF2A, however, have focused on rodents in C2C12 cell lines, which is not a suitable comparison to primary myocytes of other species because of the differences among organs and species as well as differences within the same species. For example, Snyder *et al*. (2013) reported that MEF2A knocked out in mice severely inhibited skeletal muscle regeneration [13]. Whereas, Liu *et al*. (2014) found that the deletion of MEF2A had no distinguishable effect on mice skeletal muscle histology [14]. Furthermore, the downstream genes that are regulated by MEF2A and the underlying mechanisms are far from well-studied [12], thus it is necessary to uncover the mechanisms of MEF2A in skeletal muscle myogenesis.

The aim of this study was to determine the function of MEF2A in myoblast proliferation and elucidate the regulatory mechanisms underlying the effects of MEF2A on the differentiation of myoblasts. We were able to demonstrate, for the first time, that MEF2A promoted the proliferation of skeletal muscle myoblasts. In addition, we found that MEF2A was an important transcription factor that bound to the *MyoZ2* proximal promoter and performed upstream of *MyoZ2* during myoblast differentiation. Our results reveal the roles of MEF2A in regulating bovine myoblast proliferation and differentiation *in vitro*, which can help inform theories in cattle skeletal muscle development and advance gene therapies.

## Materials and methods

### Ethics statement

A three-day old healthy Qinchuan beef cattle was used for myoblast isolation and cell culture. It was born and raised at the experimental farm of National Beef Cattle Improvement Center (Yangling, China) and slaughtered using mechanized slaughter line at Shaanxi Qinbao Animal Husbandry Development Co., Ltd. The experiments and animal care were performed according to the Regulations for the Administration of Affairs Concerning Experimental Animals (Ministry of Science and Technology, China, 2004) and approved by the Institutional Animal Care and Use Committee (College of Animal Science and Technology, Northwest A&F University, China).

### Isolation and cell culture of bovine skeletal muscle primary myoblasts

Isolation of primary skeletal muscle myoblasts was performed as previously described by Springer et al. [15]. The muscle sample was obtained from slaughter house. The limb skin was rinsed with 75% ethanol and removed with sterile sharp curved surgical scissors to expose the muscle tissue. The hind limb muscle sample was then removed into 1×PBS supplemented with 10% penicillin/streptomycin and was immediately taken into the cell culture lab. Under a stereo dissecting microscope, the muscle sample was dissected away from the blood vessel and connective tissue with sterile forceps. The muscle sample was then minced and digested with 0.25% Collagenase Ⅱ (Sigma)/0.1% Diapase Ⅱ (Roche) solution at 37°C until the mixture is a fine slurry. The cell suspension was filtered through an 80-μm cell strainer and pelleted by centrifugation for 5 minutes at 350×g. The pellets were then resuspended and seeded in 60-mm collagen-coated culture plates. The primary skeletal muscle myoblasts were cultured in complete growth medium containing Dulbecco's Modified Eagle Medium/F-12 (DMEM/F-12, Gibco), 20% fetal bovine serum (Gibco) and 1% penicillin/streptomycin. Growth medium was changed every two days and cells were passaged at 70% confluence to avoid spontaneous differentiation. For induced myogenic differentiation, cells at 70% confluence were switched to differentiation medium containing DMEM/F-12, 2% horse serum (Gibco) and 1% penicillin/streptomycin. The differentiation medium was changed every two days.

### MEF2A overexpression assay

Adenovirus carrying full length bovine *Mef2a* gene coding sequence (CDS) was generated as previously described [16]. Simply, the full length CDS sequence (Accession number: NM_001083638) was amplified and cloned into pAd-Track shuttle vector. The shutter vector was then linearized by Pme Ⅰ restriction enzyme (New England Biolabs) for recombination with pAdEasy-1 expression vector in BJ5183 competent cells. After digested with Pac Ⅰ restriction enzyme (New England Biolabs), the expression vector was purified and transfected into 293A cells to allow being packaged into adenovirus (OE-2A). Negative control (NC) adenovirus was generously provided by Changzhen Fu. (Dalian University) and Yaran Zhang. (Northwest A&F University). Viral particles were expanded in 293A cells and viral titer was determined using the end point dilution assay (Clontech).

### MEF2A interference assay

Specific short hairpin RNA (shRNA) and NC oligonucleotides for *Mef2a* mRNA (NM_001083638) were designed by using the online software: BLOCK-iT adenoviral RNAi expression system (*https://rnaidesigner.thermofisher.com/rnaiexpress/*) [17]. The specific shRNA that had the highest interference efficiency was selected through psiCHECK^TM^-Ⅱ reporter system (Promega). The specific shRNA and negative control were cloned into the pENTRTM/U6 RNAi entry vector followed by recombined with the pAd/PL-DEST^TM^ expression vector (Invitrogen). The expression vector was then transfected into 293A cells to allow to be packaged into adenoviruses (sh-2A/sh-NC). Viral particle expansion and viral titer detection were the same as OE-2A. **Table 1** shows the shRNA sequences used in this study.

**Table 1:** shRNA sense strand sequences for *Mef2a* mRNA and negative control in this study.

Name	Sense strand (5’-3’)	Loop
shRNA-571	GCAGAACCAACUCGGAUAUUG	UCAAGAG
shRNA-1193	GCCUCCACUGAAUACCCAAAG	UCAAGAG
shRNA-1219	GCAGUUCUCAAGCCACUCAAC	UCAAGAG
shRNA-1418	GCAGCACCAUUUAGGACAAGC	UCAAGAG
shRNA-NC	GUUCCACGACCAAAUCAGCUC	UCAAGAG

Note: shRNA: short hairpin RNA; “571”, “1193”, “1219”, “1418” means the position of the shRNA sequence in the mRNA region of *Mef2a*.

### Cell flow cytometry assay

Skeletal muscle myoblasts were grown in normal growth medium and passaged at 70% confluence. When grown to 60% confluence, cells in 6-well plates were infected with OE-2A or sh-2A in triplicate. 48 hours after infection, cells were washed, harvested and permeabilized by using One Step Cell Cycle Straining Kit (MultiSciences Biotech). After the nuclei was strained with propidium iodide (PI) for 30 minutes, cell cycle was detected by measuring DNA content using Flow Cytometry (FACS Calibur, BD, USA) through counting 20000 cells.

### EdU labeling assay

To detect cell proliferation, 5-ethynyl-2-deoxyuridine (EdU) assays were performed using Click-iT^®^ EdU Imaging Kit (Invitrogen) according to the manufacturer’s instructions. Cells were plated on coverslips at 60% density and treated with OE-2A or OE-NC. 48 hours after infection, cells were treated with 10μM EdU solution in normal growth medium for 1 hour. Subsequently, cells were fixed with 3.7% paraformaldehyde and permeabilized with 0.5% Triton^®^ X-100 (Sigma, USA). After incubation for 20 minutes, cells were treated with Alexa Fluor^®^ 594 azide and DAPI to stain the nuclei. Immunofluorescence images were taken by Olympus IX71 microscope (OLYMPUS).

### Vectors and plasmids

To select the specific shRNA, the full length CDS of *Mef2a* gene was cloned into psiCHECK^TM^-Ⅱ vector and the shRNA was cloned into pENTR/CMV-GFP/U6 vector. For luciferase reporter assay, the bovine *MyoZ2* proximal promoter (190bp) containing the MFE2 binding site was cloned into pGL3-Basic vector (Promega). The mutant *MyoZ2* promoter sequence was chemically synthesized (Sangon Biotech) by mutating -33bp MEF2 site TATATA to GGGGGG and was also cloned into pGL3-Basic vector. pRL-TK vector was used as internal control.

### Luciferase activity assay

The relative luciferase activity was tested 40 hours after transfection of pGL3-Basic or psiCHECK^TM^-Ⅱ vector by using Dual-Luciferase^®^ Reporter Assay System (Promega). In brief, cells were seeded in 12-well plates at a density of 1×10^6^ cells per well. Cells were treated according to the experimental design and when the cells grown to 70% confluence, cells were lysed by 1×Passive Lysis Buffer for 15 minutes at room temperature. To measure the firefly luciferase activity, 50μl Luciferase Assay Buffer Ⅱ was mixed with 20μl cell lysate followed by absorbance detection. The Renilla luciferase activity was determined by mixing 50μl 1×Stop & Glo^®^ reagent with the previous mixture. Absorbance was detected on microplate reader (TECAN, Infinite^®^ 200 PRO NanoQuant). All the luciferase assays were performed in triplicate wells and the experiment was performed 3 times.

### Cell culture immunofluorescence

The myoblasts were cultured in 6-well culture plates, fixed with 4% paraformaldehyde for 15 minutes, washed with PBS, permeabilized with 0.2% Triton X-100 for 15 minutes and then incubated in 10% (vol/vol) normal donkey serum/1% BSA (Sigma) /0.3 M glycine (Sigma) for 1h to block non-specific protein-protein interactions. For immunofluorescence, the cells were incubated with the primary antibody (diluted in 10% normal donkey serum/1% BSA/0.3 M glycine) overnight at 4°C. The cells were then washed with PBS and incubated with secondary antibody protected from light at 37°C for 1h. The nuclei were stained protected from light with DAPI (Sigma) at room temperature for 15 minutes. The antibodies were used as follows: anti-α-actinin (H-300) (1:200, Santa), and donkey anti-rabbit IgG H&L (Alexa Fluor^®^ 555) (1:1000, Abcam). DAPI was used at the final concentration of 1μg/ml. Immunofluorescence images were taken by Olympus IX71 microscope (OLYMPUS).

### siRNA transfection

Cells were seeded in 6-well plates and when cells grown to 70% confluence, siRNAs were transfected according to the standard protocol at a final concentration of 25nM. Briefly, 1.32μl X-tremeGENE HP DNA transfection reagent (Roche) and 0.66μg siRNA were diluted in Opti-MEM (Gibco) respectively for 10 minutes. The two mixtures were then mixed for another 15 minutes at room temperature to allow to form transfection reagent-siRNA complexes. The complex mixture was then added to the cell culture medium. Cells were replaced with fresh complete growth medium 8 hours later. The siRNA transfection was performed in triplicate wells and the experiment was performed with 3 repeats.

### Quantitative real time-PCR

Total RNA from myoblasts (n = 3) were isolated by Trizol reagent (Takara) according to the manufacturer’s instructions. The RNA was then applied to synthesize cDNA using PrimeScript^TM^ RT reagent Kit with gDNA Eraser (Takara). The reverse transcript reaction was performed at 37°C for 15 minutes followed by 85°C for 5s. The cDNA was then used for quantitative real time-PCR (qRT-PCR) in triplicate wells using GoTaq^®^ qPCR Master Mix (Promega) in 7500 Real Time PCR System (Applied Biosystems). The relative mRNA expression level was normalized to GAPDH. The detection of qRT-PCR was performed with 3 repeats. The experiment data were analyzed by using 2^-ΔΔCT^ method [18]. Summary information of the genes used for qRT-PCR in this study were listed in **S1 Table**.

### Western blot analysis

Cells were rinsed with PBS, digested with 0.25% trypsin (Gibco), and harvested into centrifuge tubes. Proteins were extracted using cell lysis buffer for western blot. Protein concentration was measured with BCA method (Takara). Cellular proteins were then mixed with protein loading buffer and denatured at 100°C for 10 minutes. 20μg protein samples were then subjected to 12% SDS-PAGE gel and transferred to PVDF membrane. For immune blot assay, the membrane was blocked with 5% skim milk (BD) for 2h and incubated with primary antibody diluted in blocking buffer at 4°C overnight. The membrane was then incubated with the secondary antibody protected from light at room temperature for 2h. Chemiluminescent HRP substrate (MILLIPORE) was used for taking immune blot images on BIO-RAD Molecular Imager. The images were analyzed using Image Lab software. All the immune blots were analyzed 3 times. The antibodies were used as follows: Anti-CDK1 antibody [EPR165] (Rabbit monoclonal primary antibody, 1:1000, Abcom), Anti-CDK2 antibody [E304] (Rabbit monoclonal primary antibody, 1:1000, Abcom), Anti-PCNA antibody [EPR3821] (Rabbit monoclonal primary antibody, 1:5000, Abcom), Anti-MEF2A antibody[EP1706Y] (Rabbit monoclonal primary antibody, 1:1000, Abcom), Anti-GAPDH antibody[EPR16884] (Rabbit monoclonal primary antibody, 1:10000, Abcom), Anti-β-actin antibody (Rabbit Polyclonal primary antibody, 1:10000, Novus), Anti-α-actinin Antibody (H-300) (Rabbit Polyclonal primary antibody, 1:200, Santa Cruz Biotechnology), Goat anti-IgG H&L (HRP) (1:2000, Abcom), Donkey Anti-Rabbit IgG H&L (Alexa Fluor^®^ 555, 1:2000, Abcom).

### Statistical analysis

All data are presented as mean ± SEM. Statistically significant differences between two groups were analyzed using Independent-samples t-test, and among three or more groups were analyzed by one-way analysis of variance (ANOVA). *P* < 0.05 was statistically significant [13].

## Results

### The *Mef2* genes expression patterns during myoblast differentiation

**Fig 1:**
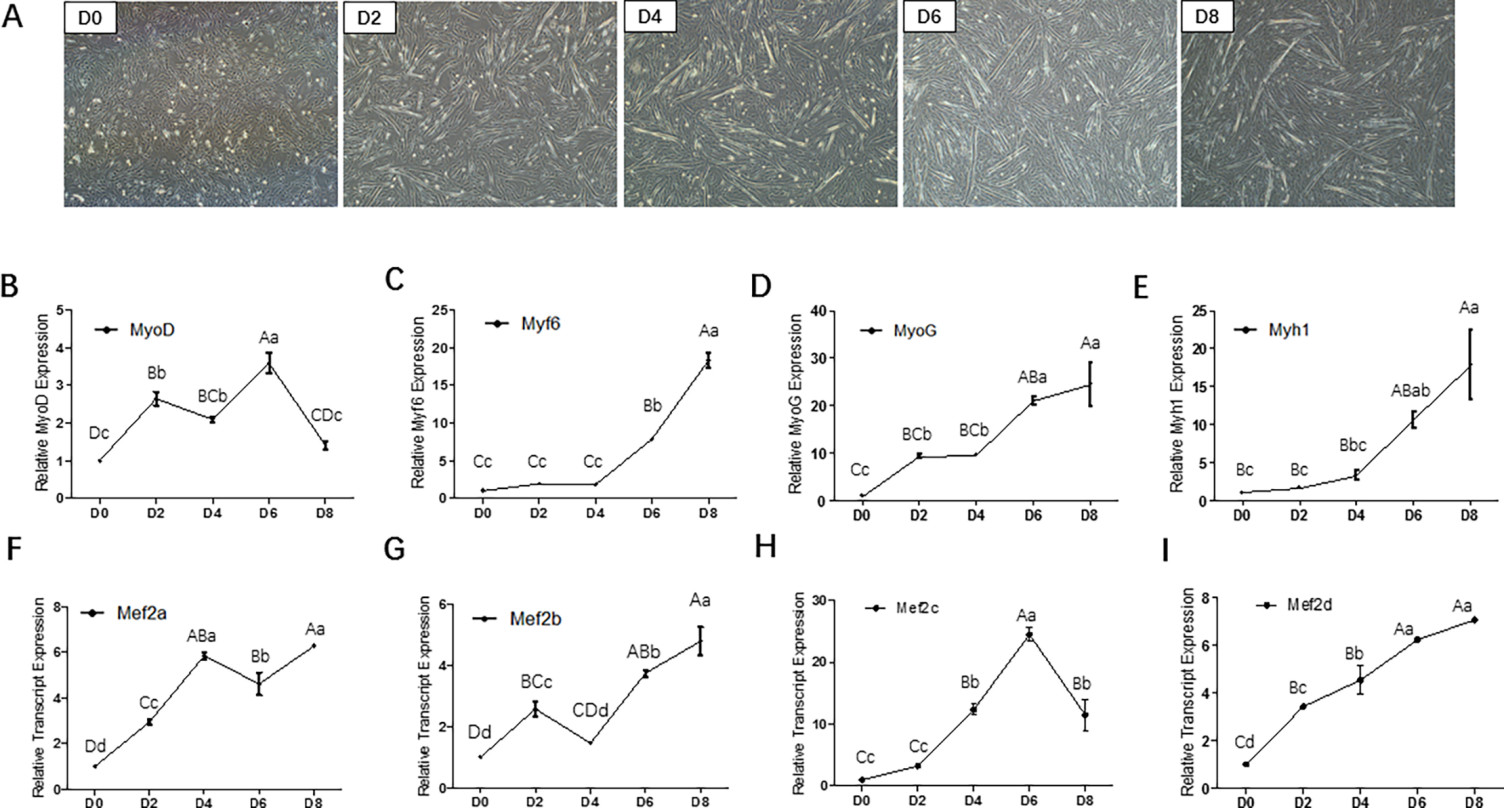
*Mef2* mRNA expression patterns in bovine myocytes. (A) Cell culture of isolated bovine skeletal muscle primary myoblast and induction of myogenesis *in vitro* (OLYMPUS IX71 40×). (B~E) Relative mRNA expression of *MyoD*, *Mrf4*, *MyoG* and *Myh1*. (F~I) Relative mRNA expression of *Mef2a*, *Mef2b*, *Mef2c* and *Mef2d*. Error bars represent s.e.m. Different lowercases among different columns represent *P* < 0.05. Different uppercases among different columns represent *P* < 0.01.

To perform this study, primary bovine myoblasts were isolated from hind limb muscle and induced to myogenic differentiation. Forty-eight hours after myogenic induction, myoblasts began to form short myotubes (**Fig 1A**). As the culture continued, cells gradually elongated and most of the cultured cells fused into myotubes. The mRNA expression levels of the key myogenic transcription factors including *MyoD*, *Mrf4*, *MyoG* and *Myh1* were measured at 0, 2, 4, 6 and 8 days after induction. The results showed that the mRNA levels of *MyoD* was higher in the early stage of differentiation and then gradually decreased (**Fig 1B**). In contrast, the mRNA levels of *Mrf4*, *MyoG* and *Myh1* were continuously elevated during myoblast differentiation (**Fig 1C–1E**). These results indicated that the isolated primary myoblasts were satisfactory for the subsequent experiments.

To investigate the relationships between *Mef2* and myoblast differentiation, the mRNA levels of *Mef2a*, *Mef2b*, *Mef2c* and *Mef2d* were also measured at 0, 2, 4, 6 and 8 days after induction. The mRNA levels of the four genes were all up-regulated during myoblast differentiation compared to that of the non-differentiated myoblasts (**Fig 1F–1I**). Moreover, the expression patterns of the four genes differed from each other. *Mef2a* was one of the first genes to exhibit differential expression in the time-course analysis. These differences suggest that the four genes of the MEF2 family perform distinct roles in skeletal muscle development. The time dependent expression pattern may reflect a specific role for MEF2A in bovine skeletal muscle myogenesis.

### Construction of recombinant adenovirus to overexpress or interfere MEF2A

**Fig 2:**
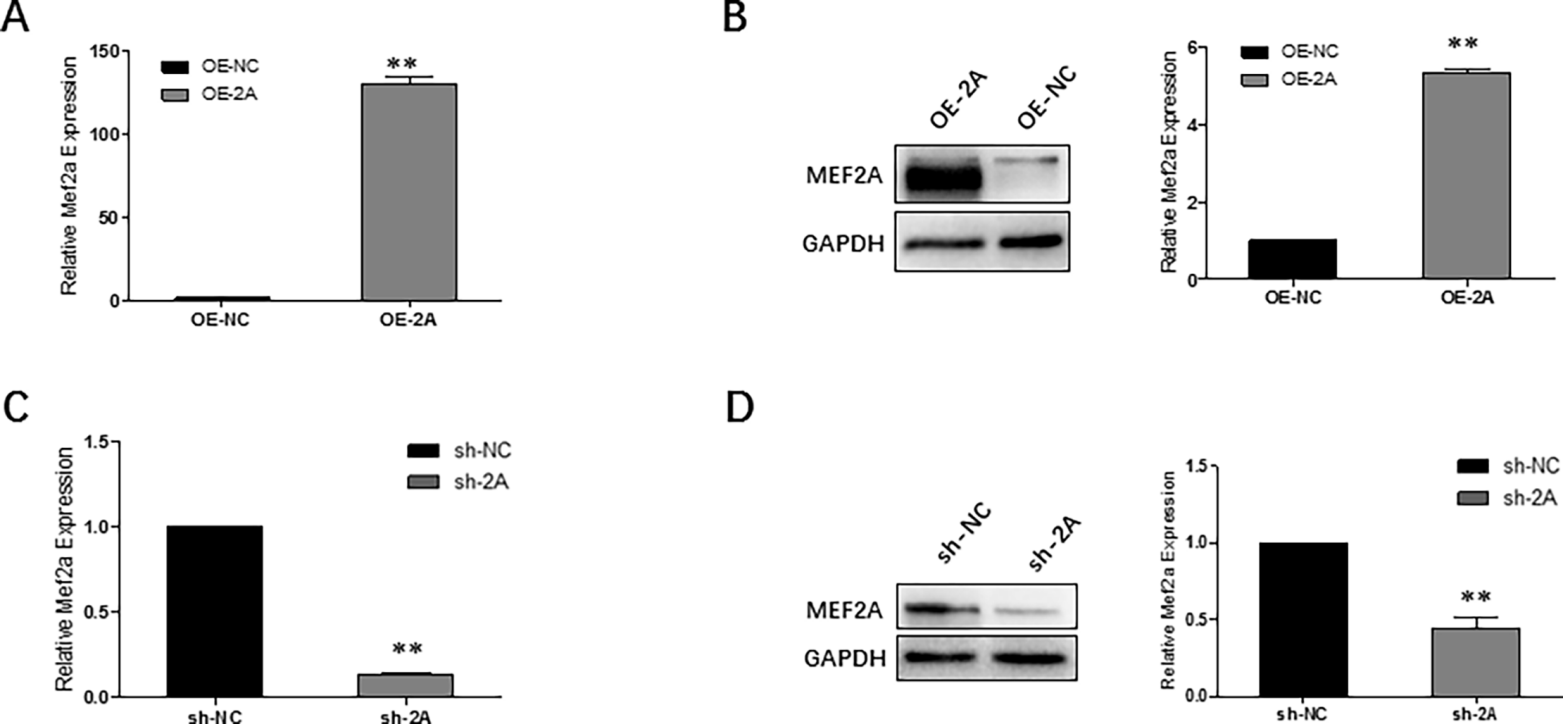
Construction of recombinant adenovirus to overexpress or interfere MEF2A. (A~B) OE-2A efficiently elevated mRNA expression level (A) and protein expression level (B)of Mef2a in myoblasts. (C~D) sh-2A efficiently interfered MEF2A mRNA expression level (C) and protein expression level (D) in myoblasts. Error bars represent s.e.m. *P < 0.05; **P < 0.01.

Specific adenovirus either to overexpress or knockdown MEF2A were generated in 293A cells. Adenovirus carrying full length bovine *Mef2a* CDS was successfully packaged within 8 days (**S1A Fig**). Cells were infected at a multiplicity of infection (MOI) of 50. The expression efficiency of OE-2A was examined through infection of skeletal muscle myoblasts. The relative mRNA expression level of *Mef2a* increased nearly 150 times in OE-2A infected cells compared to the control group (**Fig 2A**). The MEF2A protein expression level also significantly up-regulated in OE-2A infected myoblasts (**Fig 2B**).

Specific shRNA used to inhibit MEF2A expression was subsequently designed (**S1B Fig**). To select the specific shRNA with the highest interference efficiency, psiCHECK^TM^-Ⅱ luciferase reporter assay was carried out in 293A cells. As shown in **S1C Fig**, sh-1219 was selected as the specific sh-RNA to interfere MFE2A expression. Adenovirus carrying the selected shRNA were successfully packaged within 15 days (sh-2A and sh-NC) and viral particles were expanded in 293A cells (**S1D Fig**). Primary skeletal muscle myoblasts were infected with sh-2A or sh-NC at MOI of 50. As shown in **Fig 2C**, the shRNA had efficiently reduced mRNA expression level *Mef2a* gene nearly up to 87%. Although sh-NC still had nearly 20% knockdown efficiency, the final *Mef2a* transcript expression level showed no differences between sh-NC and control group (data not shown). Western blot analysis showed that MEF2A protein expression also significantly decreased in sh-2A infected myoblasts (**Fig 2D**).

### MEF2A promotes myoblast proliferation by triggering cell cycle progression

**Fig 3:**
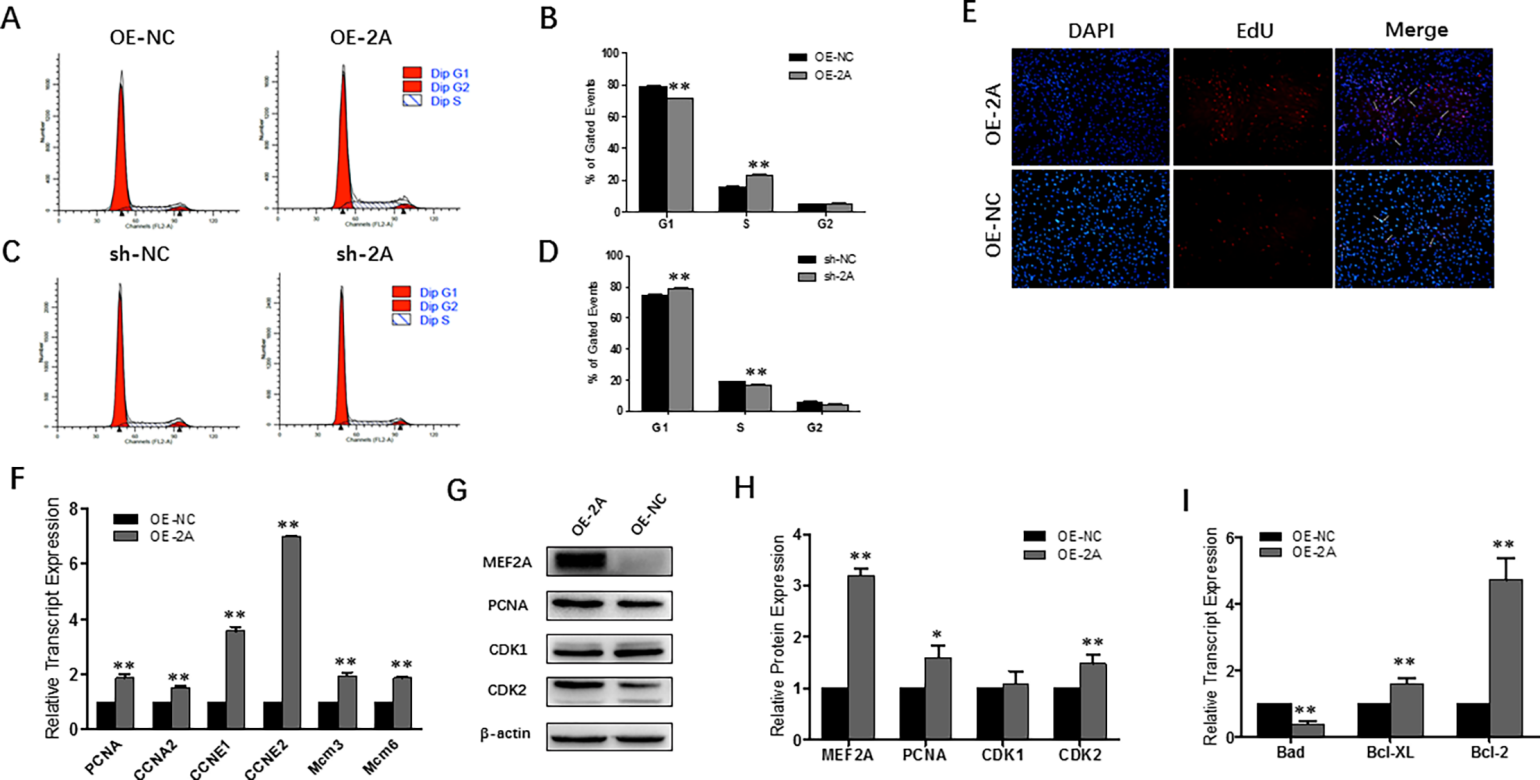
MEF2A promotes myoblast proliferation through triggering cell cycle progression. (A~B) Flow cytometric measurement of DNA content using propidium iodide (PI) staining in OE-2A/OE-NC treated proliferating myoblast. (C~D) Flow cytometric measurement of DNA content using propidium iodide (PI) staining in sh-2A/sh-NC treated proliferating myoblast. (E) Images of the EdU assay: DAPI staining is shown in blue and EdU staining is in red (OLYMPUS IX71 100×). (F) Relative mRNA expression of cell cycle genes: *PCNA*, *CCNA2*, *CCNE1*, *CCNE2*, *Mcm3* and *Mcm6*. (G~H) Western blot and protein expression analysis of PCNA, CDK1 and CDK2 showed that MFE2A activated CDK2 expression but not CDK1 expression. (I) Relative mRNA expression of pro-apoptotic gene (*Bad*) and pro-survival genes (*Bcl-2* and *Bcl-XL*) at early apoptotic stage. Error bars represent s.e.m. *P < 0.05; **P < 0.01.

Overexpression of MEF2A in myoblasts induced a noticeable decrease in G1 phase cell counts and increased S phage cell counts (**Fig 3A and 3B**). While suppressing MEF2A expression, it showed the opposite effects (**Fig 3C and 3D**) in that cells were blocked in the G1 phase in sh-2A infected myoblasts. The apparent transition from the G1 phase to S phase in OE-2A infected myoblasts suggested a positive effect of MEF2A on myoblast proliferation. To confirm whether increased cell cycle activity was associated with increased DNA synthesis, EdU incorporation assay was performed. Results showed that overexpression of MEF2A induced a noticeable increase in EdU^+^ myoblasts (**Fig 3E**).

Proliferating cell nuclear antigen (PCNA) is an essential cofactor in DNA replication. RT-PCR and Western blot analysis showed that PCNA was up-regulated upon overexpression of MEF2A (**Fig 3F–3H**). We also found that the mRNA levels of cell cycle genes, including *CCNA2*, *CCNE1*, *CCNE2*, *Mcm3* and *Mcm6*, were all up-regulated in OE-2A treated myoblasts (**Fig 3F**). In vertebrates, cyclin E binds to and activates CDK2 and then promotes the G1 to S phase transition. Cyclin A binds to CDK1 and activates G2 phase [19]. In this study, we found that overexpression of MEF2A increased CDK2 but not CDK1 protein expression (**Fig 3G and 3H**), resulting in promoted G1 to S phase transition. *Bad* is a pro-apoptotic gene and *Bcl-XL*, *Bcl-2* are pro-survival genes that functions at early apoptotic stage in mitochondria. In this study, mRNA expression of these genes was also examined in OE-2A infected myoblasts. Theas results showed that MEF2A overexpression didn’t induce myoblast apoptosis or cell death.

### MEF2A knockdown inhibits myoblast differentiation

**Fig 4:**
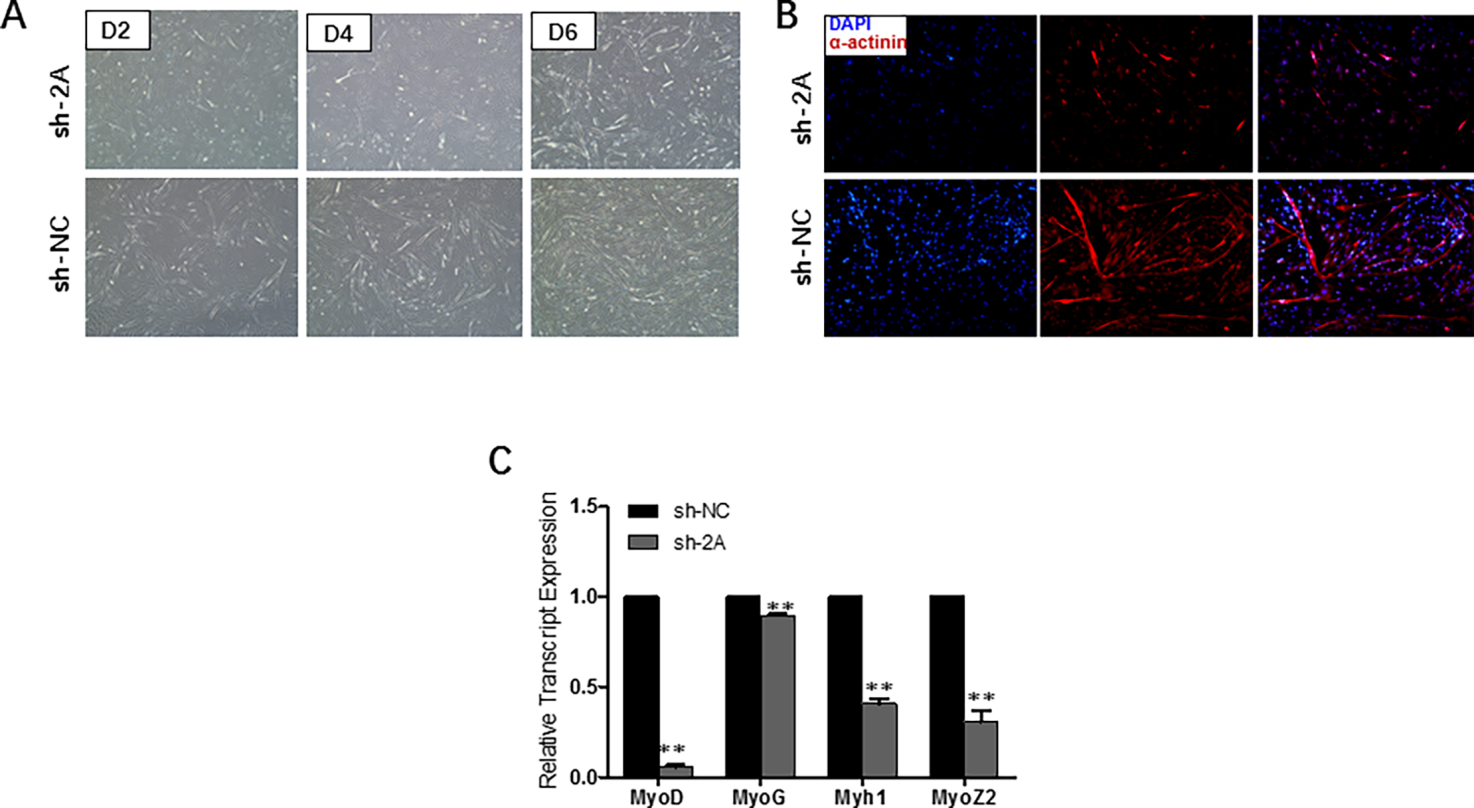
MEF2A knockdown inhibits myoblast differentiation. (A) Morphological changes of differentiating bovine skeletal primary myoblasts at 2-day (D2), 4-day (D4) and 6-day (D6) after infection of sh-2A and sh-NC (OLYMPUS IX71 40×) (B) Images of immunofluorescence assay at differentiation day 6 treated with sh-2A or sh-NC (OLYMPUS IX71 100×). DAPI staining is shown in blue and α-actinin is shown in red. (C) Relative mRNA expression levels of myoblast differentiation marker genes (*MyoD*, *Mrf4*, *MyoG* and *Myh1*) significantly reduced in sh-2A treated cells. Error bars represent s.e.m. *P < 0.05; **P < 0.01.

To investigate the function of MEF2A in regulating myoblast differentiation, MEF2A interference assay was carried out by using primary myoblasts. As shown in **Fig 4A**, MEF2A knocked down in differentiating myoblasts resulted in severely impaired myotube formation. During myogenic differentiation, sh-2A infected myocytes exhibited poor differentiation potential from D0 to D6. The amount of α-actinin^+^ myocytes at D6 of differentiation decreased significantly in MEF2A interfered myoblasts (**Fig 4B**). Upon determining the roles of MEF2A in myoblast differentiation, the relative transcript expression levels of MRFs and myosin were also examined because MRFs play essential roles in myogenic determination and terminal differentiation process [20, 21]. The mRNA expression levels of *MyoD*, *Mrf4*, *MyoG*, *Myh1* and *MyoZ2* were significantly down-regulated in MEF2A interfered myoblasts. In this process, MEF2A affects both the early myogenic determination and the terminal differentiation stages.

### MEF2A transcriptionally regulates the *MyoZ2* proximal promoter in bovine myoblasts

**Fig 5:**
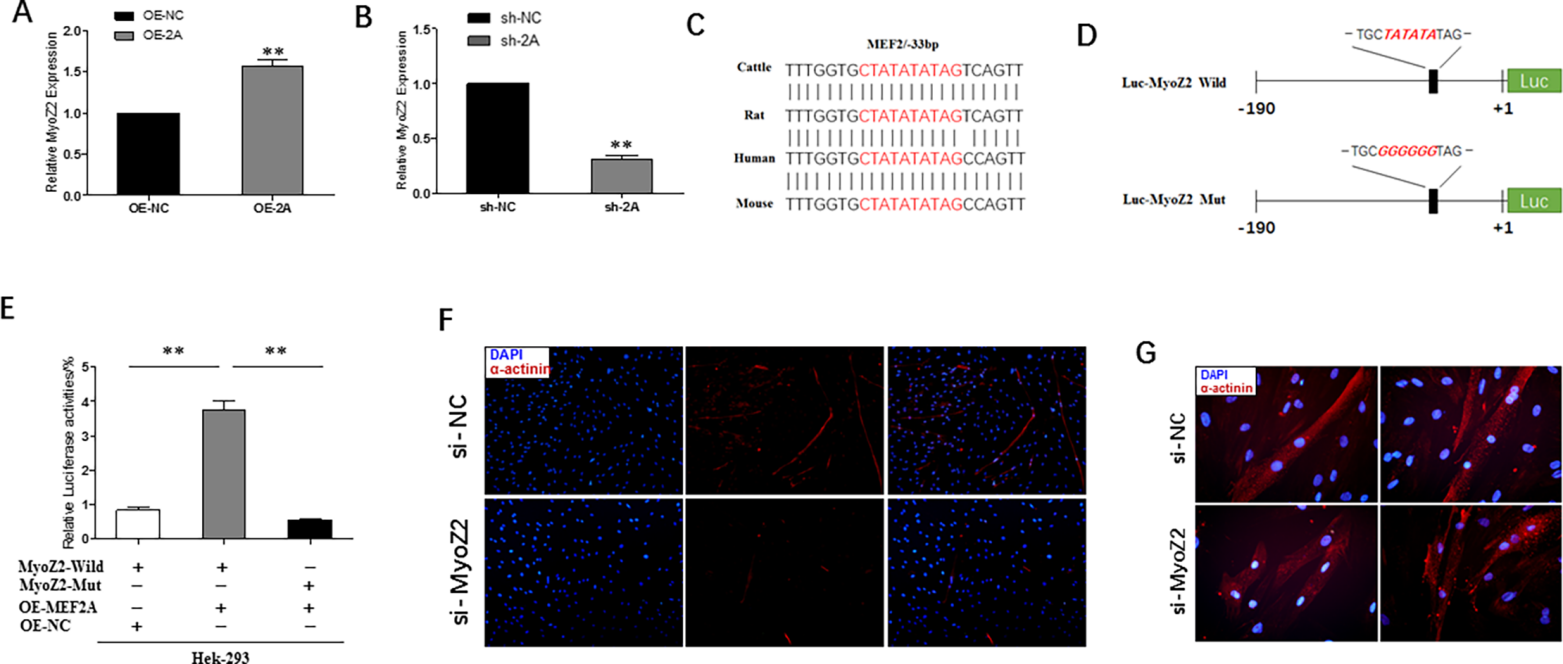
*MyoZ2* transcriptional activity is regulated by MEF2A and silencing *MyoZ2* inhibited myoblast differentiation. (A~B) Relative mRNA expression analysis of *MyoZ2* showed that *MyoZ2* was closely co-expressed with *Mef2a*. (C) Sequence alignments of MEF2 transcription binding site in the *MyoZ2* promoter among cattle, rat, human and mouse was highly conserved. (D) Structure of *MyoZ2* luciferase reporter vector with wild or mutant -33 bp MEF2 binding site. The mutant sequence was marked in italic red. (E) Luciferase analysis showed that MEF2A could efficiently promote *MyoZ2* transcriptional activity. (F~G) Images of immunofluorescence assay at differentiation day 6 transfected with *MyoZ2* specific siRNA or negative control siRNA (F: OLYMPUS IX71 100×; G: OLYMPUS IX71 400×). Error bars represent s.e.m. *P < 0.05; **P < 0.01.

MYOZ2 is a sarcomeric calcineurin-binding protein residing in the Z-disk that plays a crucial role in myofiber formation and human hypertrophic cardiomyopathy [22–24]. Previous studies from our laboratory predicted that MEF2A was an important transcription factor that binds to the *MyoZ2* proximal promoter and might regulate *MyoZ2* transcriptional activity. In the present study, we found that *MyoZ2* closely co-expressed with *Mef2a* (**Fig 5A and 5B**). Sequence alignment of the bovine *MyoZ2* promoter revealed that the only -33bp MEF2 binding site, C/T TA(A/T)4TA G/A [25, 26], and its flanking sequences were nearly completely conserved among human, mouse, rat and bovine genomes (**Fig 5C**). To investigate whether MEF2A could regulate *MyoZ2* transcriptional activity, *MyoZ2* luciferase reporter vector with wild or mutant -33bp MEF2 binding site was generated and transfected into 293A cells (**Fig 5D**). The results showed that MEF2A could activate the *MyoZ2* proximal promoter that harbored the MEF2 transcription factor binding site. 293A cells co-transfected with OE-2A and the *MyoZ2* promoter resulted in two-fold higher levels of luciferase activity compared to the control group (**Fig 5E**). On the other hand, mutation of MEF2 binding site significantly reduced *MyoZ2* transcriptional activity (**Fig 5E**). These results indicated that MEF2A promoted *MyoZ2* expression by activating the promoter region.

### Silencing *MyoZ2* in myoblasts results in impaired myotube formation

To investigate the role of *MyoZ2* in myoblast differentiation, *MyoZ2* silencing assay were performed by using specific siRNA. Consistent with the inhibitory effect of MEF2A, interference of *MyoZ2* also resulted in severely impaired myotube formation (**Fig 5F**). In detail, we could see the deficiency effects more morphologically from **Fig 5G**. In the control group, there were more myofibers and myotubes were much longer. Fusion of myotubes was much more better with more nuclei in each myofiber. However, in siRNA treated myoblasts, myotubes were shorter and fragmented. The majority of the myoblasts did not fuse into myotubes and only few myoblasts were poorly fused. In addition, expression of α-actinin was significantly lower due to the abnormal distribution of α-actinin protein and myotubes compared to that of the control. These results indicated that *MyoZ2* was also likely involved in myotube formation and performed downstream of MEF2A in regulating myoblast differentiation.

## Discussion

Skeletal muscle myoblast proliferation and differentiation are very important in determining muscle growth and muscle quality. In the present study, we investigated the roles of *Mef2a* in regulating the proliferation and differentiation of bovine skeletal muscle primary myoblast. There were two major findings in our study. First, we found that MEF2A expression promoted myoblast proliferation by triggering cell cycle progression through up-regulation of CDK2 expression. This is the first evidence that MEF2A is required for skeletal muscle myoblast proliferation. Second, interference of MEF2A expression in myoblasts blocked myogenic differentiation by down-regulation of *MyoZ2* transcriptional activity.

Multiple lines of evidence have suggested that the genes of the MEF2 family play pivotal roles in embryonic development [7, 27], skeletal muscle fiber formation [28, 29], and muscle or cardiac disease [4, 30–32]. To some extent, gene expression patterns of MEF2 genes can reflect their functions in the relevant tissue or cells. In rodents, the four MEF2 genes are all up-regulated in myogenesis [13]. In skeletal muscle development, *Mef2a*, *Mef2c* and *Mef2d* play more important roles than *Mef2b*. During embryonic development, *Mef2a* expresses later than *Mef2c* [27]. During skeletal muscle regeneration, *Mef2a* is one of the earliest genes that can be detected, whereas *Mef2c* and *Mef2d* expression occurs much later [13]. In our study, we also observed a general trend of the gradual expression of the four MEF2 genes. However, expression of *Mef2a* occurs much earlier than expression of *Mef2c* although *Mef2c* exhibits obviously differential expression pattern. This notion suggests that *Mef2a* may play different roles compared to other genes of the MEF2 family in skeletal muscle.

MEF2A has been known to regulate proliferation, survival, and apoptotic pathways in a variety of specialized cell types, such as neurons, cardiomyocytes, immune cells, vascular smooth muscle cells and endothelial cells [7, 9, 33–35]. Until now, it was unknown whether MEF2A could regulate skeletal muscle myoblast proliferation. Generally, the prototypical eukaryotic cell cycle is divided into four phases that are tightly controlled by CDK-Cyclin complexes [36]. During mammalian cell cycle, CDK4 and CDK6 together with D-type cyclins promote the transition from the gap 0 (G0) phase to gap 1 (G1) phase. Subsequently, CDK2 controls entry into the S phase in complex with Cyclin E and Cyclin A. Cdk1, in conjunction with Cyclin A and Cyclin B, then controls the entry and progression through the M phase [37]. The major checkpoints are G1-S, G2-M and progression into anaphase. In our study, by detecting cell cycle with flow cytometry, we found that MEF2A expression promoted transition of bovine skeletal myoblasts from the G1 to S phase, while MEF2A inhibition arrests the cell cycle in the G1 phase. This positive effect activates CDK2 but not CDK1 protein expression. These results differ from what Liu (2014) reported that MEF2 is dispensable in mouse satellite cell proliferation [14]. However, we believe their result is not very precise because the methods used were too subjective. In addition, their EdU labeling time was too long and likely allowed some cells to have finished one cell cycle and entered into another. Although further investigations on how MEF2A activates myoblast proliferation is needed, we conclude that MEF2A is likely a positive regulator in the process of bovine myoblast cell cycle.

At present, the role of MEF2A in skeletal muscle cell differentiation has been extensively studied, and it has been found that MEF2A nearly had no effect on skeletal muscle development [14, 38]. However, contradictory results have also been reported. Estrella *et al*. and Synder *et al*. revealed that MEF2A was quite necessary for mice skeletal muscle differentiation and regeneration [12, 13]. They explained that the phenotypic difference was due to radically different MEF2 temporal expression in C2C12 cells and developmental stages [13]. In our study, we found that expression of MEF2A was necessary for bovine skeletal muscle myotube formation. What should be noticed was that all these studies mentioned above were performed under different developmental stages and experimental conditions (in vitro or in vivo) in different animal models even though on the same organ: skeletal muscle. Thus, the functions of MEF2A in skeletal muscle is time-, dose-, environment- and species-dependent. *MyoD* is a myogenic determination gene that is expressed prior to differentiation. *Mrf4* acts as both a determination and a differentiation factor, whereas *MyoG* is a terminal differentiation gene that expresses later [39]. Meanwhile, myosin is a superfamily that constructs myofibers. Myosin disfunction can result in severe muscular dystrophy and muscle diseases [40, 41]. Overall, the impairment differentiation effect of interfering MEF2A in proliferating myoblasts occurred from the determination stage to the terminal differentiation stage.

*MyoZ2* is an important Z-disk gene in the sarcomere structure that is required for normal myotube formation as well as muscle development [42]. It is also an inhibitor of calcineurin (also called protein phosphatase 2B, PP2B) that regulates myofiber type and muscle development through the calcineurin-NFAT signaling pathway [43]. Meanwhile, MEF2A is an important transcription factor in CaMK-HDACs, calcineurin and MAPK pathway [8, 9]. To data, there is nearly no evidence that clarifies whether MEF2A interacts with *MyoZ2* in myoblasts. However, in our study, we noticed a tight co-expression of *MyoZ2* and MEF2A in bovine skeletal muscle myoblasts. We also found that *MyoZ2* transcriptional activity was directly regulated by MEF2A. Moreover, a ChIP-seq study found that *MyoZ2* was immune precipitated by MEF2A antibody [44]. However, further investigation is needed to conclusively determine whether MEF2A directly interacts with *MyoZ2*. Cardiac hypertrophy is the abnormal muscle development. Current evidence suggests that overexpression of *MyoZ2* can inhibit calcineurin-dependent signaling and result in overload-induced cardiac hypertrophy as well as arterial hypertension [45, 46]. In the present study, *MyoZ2* may function through the similar mechanisms in skeletal myoblast differentiation. Notably, we found that *MyoZ2* inhibition also reduced α-actinin expression which caused the abnormal protein distribution in myotubes. Therefore, while α-actinin forms the major components of the contractile apparatus at the Z-disk and regulates dystrophin [47, 48], it is likely that *MyoZ2* inhibition induced myoblast differentiation defect is closely related to Z-disk protein activity.

**Fig 6:**
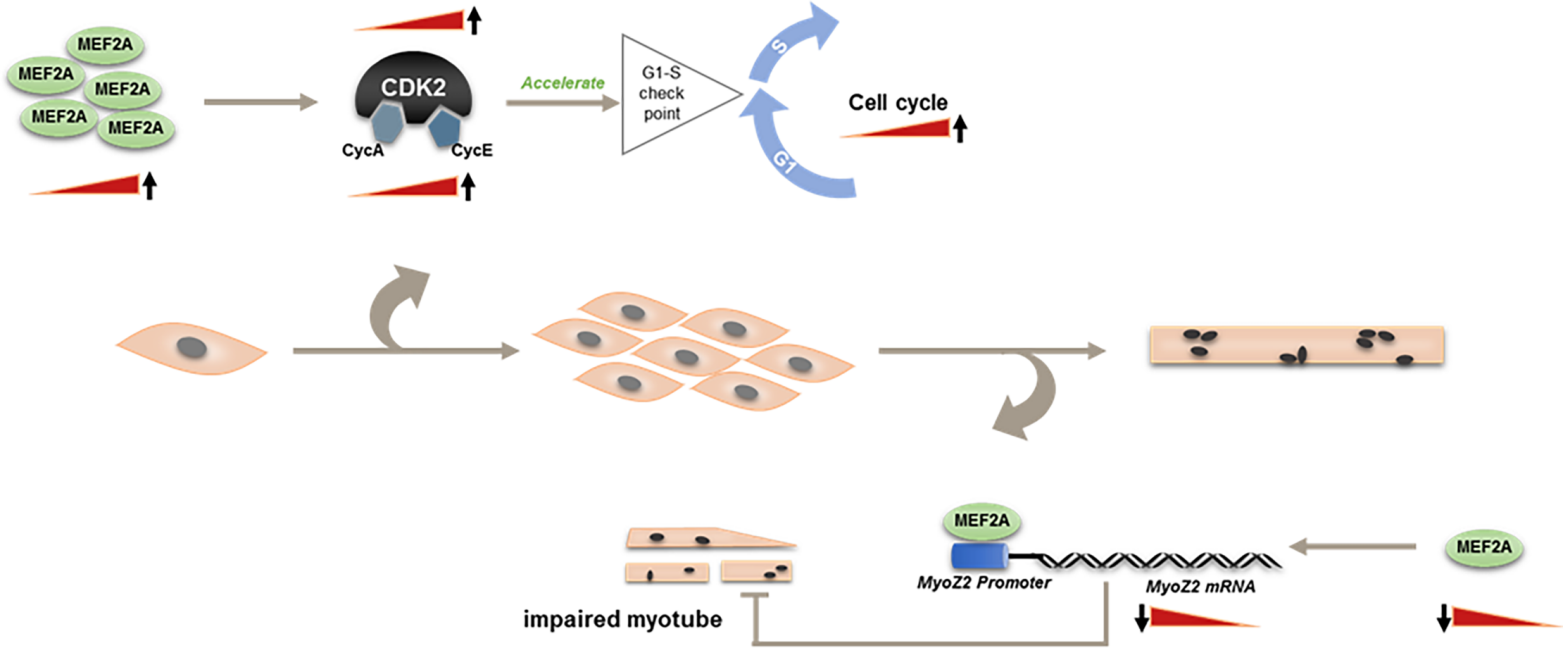
Mechanisms of MEF2A in regulating myogenesis of bovine skeletal muscle primary myoblasts. MEF2A acts as a positive regulator in myoblast proliferation. It can promote cell cycle transition from G1 to S phase by activating CDK2. In addition, knockdown of MEF2A in myoblasts inhibits myogenic differentiation via transcriptionally regulating *MyoZ2* expression.

In conclusion, we report that MEF2A is a positive regulator in the proliferation and differentiation of bovine skeletal muscle primary myoblasts. MEF2A promotes myoblast proliferation by triggering cell cycle progression by activating CDK2 expression and regulates myoblast differentiation through transcriptional regulation of *MyoZ2* (**Fig 6**). This study sheds some light on the roles of MEF2A in regulating bovine myoblast proliferation and differentiation *in vitro* and can help inform theories in cattle skeletal muscle development and improve gene therapies.

## Supporting information

S1 Fig Construction of recombinant adenovirus to overexpress or interfere MEF2A. (A) Recombinant adenovirus carrying full length bovine *Mef2a* CDS (OE-2A) was packaged within 8 days in 293A cells (40×). (B) Locations of sh-RNA that are specific for MEF2A. The four sh-RNAs are separated from each other vary from exon 4 to exon 10. (C) All the four specific sh-RNAs could significantly reduce MEF2A transcription efficiency. (D) Recombinant adenovirus carrying specific shRNA (sh-2A) and negative control shRNA (sh-NC) were packaged within 15 days in 293A cells (40×). Error bars represent s.e.m. Different lowercases among different columns represent *P* < 0.05. Different uppercases among different columns represent *P* < 0.01.
(TIF)

S1 Table Summary information of the genes used for qRT-PCR in this study. (DOCX)
