# Supplementary figures and images for: Myocyte enhancer factor 2A promotes proliferation and its inhibition attenuates myogenic differentiation via myozenin 2 in bovine skeletal muscle myoblast
Source: PLoS One. 2018 Apr 26;13(4):e0196255. doi: 10.1371/journal.pone.0196255 (PMC5919640; doi:10.1371/journal.pone.0196255)

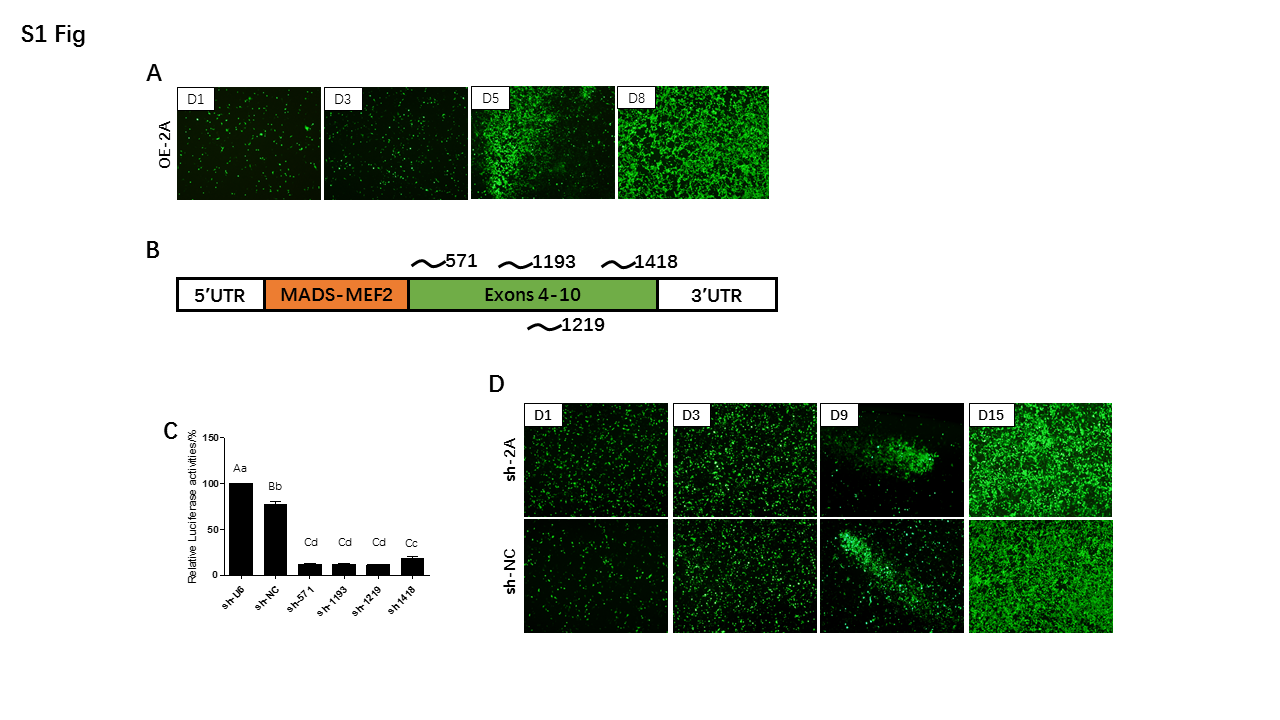

Supplement: S1 Fig — (A) Recombinant adenovirus carrying full length bovine Mef2a CDS (OE-2A) was packaged within 8 days in 293A cells (40×). (B) Locations of sh-RNA that are specific for MEF2A. The four sh-RNAs are separated from each other vary from exon 4 to exon 10. (C) All the four specific sh-RNAs could significantly reduce MEF2A transcription efficiency. (D) Recombinant adenovirus carrying specific shRNA (sh-2A) and negative control shRNA (sh-NC) were packaged within 15 days in 293A cells (40×). Error bars represent s.e.m. Different lowercases among different columns represent P < 0.05. Different uppercases among different columns represent P < 0.01. (TIF) [file pone.0196255.s001.tif]
