# Supplementary material for: Myocyte enhancer factor 2A promotes proliferation and its inhibition attenuates myogenic differentiation via myozenin 2 in bovine skeletal muscle myoblast
Source: PLoS One. 2018 Apr 26;13(4):e0196255. doi: 10.1371/journal.pone.0196255 (PMC5919640; doi:10.1371/journal.pone.0196255)
Supplement: S1 Table — (DOCX) [file pone.0196255.s002.docx]

**S1 Table**. **Summary information of the genes used for qRT-PCR in this study**

| Gene | GenBank Transcript ID | Purpose | Forward primer sequence (5’-3’) | Reverse primer sequence (5’-3’) | Product size |
| --- | --- | --- | --- | --- | --- |
| *Mef2a* | NM_001083638 (*Bos taurus*) | Gene expression | AATGAACCTCACGAAAGCAGAAC | TTAGCACATAGGAAGTATCAGGGTC | 106bp |
| *Mef2b* | NM_001145793 (*Bos taurus*) | Gene expression | AGCAGACAAAGGGCACTCCA | TTTGGTCCAGAATGCGTGAG | 76bp |
| *Mef2c* | NM_001046113 (*Bos taurus*) | Gene expression | CCTGATGCAGACGATTCAGTAG | AAAGTTGGGAGGTGGAACAG | 123bp |
| *Mef2d* | NM_001205178 (*Bos taurus*) | Gene expression | TGTGTCTCTCAGCAACCTAATC | ACACTGGTTCCGACTTGATG | 104bp |
| *MyoD* | NM_001040478 (*Bos taurus*) | Gene expression | AACCCCAACCCGATTTACC | CACAACAGTTCCTTCGCCTCT | 196bp |
| *Mrf4* | NM_181811 (*Bos taurus*) | Gene expression | GTGATAACTGCCAAGGAAGGAG | CGAGGAAATGCTGTCCACGA | 93bp |
| *MyoG* | NM_001111325 (*Bos taurus*) | Gene expression | GGCGTGTAAGGTGTGTAAG | CTTCTTGAGTCTGCGCTTCT | 85bp |
| *Myh1* | NM_174117 (*Bos taurus*) | Gene expression | GGCAGGGTCTTTGATTGGG | TTAGGGTCCGCCACGAAGA | 172bp |
| *PCNA* | NM_001034494 (*Bos taurus*) | Gene expression | CCTTGGTGCAGCTAACCCTT | TTGGACATGCTGGTGAGGTT | 94bp |
| *CCNA2* | NM_001075123 (*Bos taurus*) | Gene expression | GCAGCCTTTCATTTAGCACTCT | ATTGACTGTTGTGCGTGCTG | 155bp |
| *CCNE1* | NM_001192776 (*Bos taurus*) | Gene expression | CGATGTCTCTGTTCGCTCCA | CCACACTGGCTTCTCACAGT | 108bp |
| *CCNE2* | NM_001015665 (*Bos taurus*) | Gene expression | GCTTATGTCACTGATGGTGCTTG | TTAGCCAGGAGATGACCGTTAC | 122bp |
| *Mcm3* | NM_001013586 (*Bos taurus*) | Gene expression | TGGTGACGCTATGCCTCTTG | GGTCATCAGGGCTGAAGTTGG | 74bp |
| *Mcm6* | NM_001046234 (*Bos taurus*) | Gene expression | TCTTCATGGAGGATTACAGTGCG | CGAGATTGACATCAGGTGTTTCC | 166bp |
| *MyoZ2* | NM_001015676 (*Bos taurus*) | Gene expression | AACAGAGTTGCCACCCCATT | CCATCCCTTCGGAGTCCTATT | 162bp |
| *Bad* | NM_001035459 (*Bos taurus*) | Gene expression | TCCCAGAGTTTGAGCAGAGTG | TTAGCCAGTGCTTGCTGAGAC | 108bp |
| *Bcl-2* | NM_001166486 (*Bos taurus*) | Gene expression | ATGTGTGTGGAGAGCGTCAA | ATACAGCTCCACAAAGGCGT | 138bp |
| *BCL-XL* | NM_001077486 (*Bos taurus*) | Gene expression | CACTGTGCGTGGAAAGCGTA | GCTGCATTGTTCCCGTAGAG | 154bp |
| *GAPDH* | NM_001034034 (*Bos taurus*) | Gene expression | AGTTCAACGGCACAGTCAAGG | ACCACATACTCAGCACCAGCA | 124bp |
